# Review of Recent Advances in Thiazolidin-4-One Derivatives as Promising Antitubercular Agents (2021–Present)

**DOI:** 10.3390/molecules30102201

**Published:** 2025-05-17

**Authors:** Wiktoria Drzał, Nazar Trotsko

**Affiliations:** 1 Students Research Group, Department of Organic Chemistry, Medical University of Lublin, 4A Chodzki Street, 20-093 Lublin, Poland; 63752@student.umlub.pl; 2 Department of Organic Chemistry, Medical University of Lublin, 4A Chodzki Street, 20-093 Lublin, Poland

**Keywords:** thiazolidin-4-one, thiazolidine-2,4-dione, rhodanine, 2-iminothiazolidin-4-one, antitubercular activity, structure–activity relationships, *Mycobacterium tuberculosis*

## Abstract

Tuberculosis (TB) remains one of the leading causes of mortality worldwide, exacerbated by the emergence of multidrug-resistant (MDR) and extensively drug-resistant (XDR) Mycobacterium tuberculosis strains. In the pursuit of novel therapeutic strategies, thiazolidin-4-one derivatives have gained significant attention due to their structural diversity and broad-spectrum biological activities. This review provides a comprehensive summary of recent advances (2021–present) in the synthesis, structure–activity relationship (SAR), and mechanisms of action of thiazolidin-4-one derivatives as promising antitubercular agents. A detailed discussion of synthetic pathways is presented, including classical and multi-component reactions leading to various subclasses such as thiazolidine-2,4-diones, rhodanines, and pseudothiohydantoins. The SAR analysis highlights key functional groups that enhance antimycobacterial activity, such as halogen substitutions and heterocyclic linkers, while molecular docking and in vitro studies elucidate interactions with key *Mtb* targets including InhA, MmpL3, and DNA gyrase. Several compounds demonstrate potent inhibitory effects with MIC values lower than or comparable to first-line TB drugs, alongside favorable cytotoxicity profiles. These findings underscore the potential of thiazolidin-4-one scaffolds as a valuable platform for the development of next-generation antitubercular therapeutics.

## 1. Introduction

Tuberculosis (TB) remains one of the world’s deadliest infectious diseases, caused by *Mycobacterium tuberculosis* (*Mtb*), and responsible for millions of deaths annually. As reported in the Global Tuberculosis Report 2024, tuberculosis caused an estimated 1.25 million deaths worldwide in 2023—including 1.09 million among HIV-negative individuals and 161,000 among people living with HIV [1]. Despite the availability of first-line anti-TB drugs such as isoniazid, rifampicin, pyrazinamide, and ethambutol, the emergence of multidrug-resistant (MDR) and extensively drug-resistant (XDR) *Mtb* strains presents a major global health challenge. These alarming trends highlight the urgent need for new therapeutic agents with novel mechanisms of action to combat resistant strains and shorten treatment regimens.

Among various heterocyclic scaffolds investigated for their biological potential, thiazolidin-4-one derivatives have garnered significant attention due to their structural versatility and broad-spectrum pharmacological activities, including antimicrobial [2,3,4], anticancer [5,6,7,8], anti-inflammatory [9,10], antiprotozoal [11,12], and antitubercular effects [13]. Notably, several recent studies have demonstrated that thiazolidin-4-one-based compounds exhibit promising inhibitory activity against *Mtb*, often comparable or superior to the existing drugs, while targeting key bacterial enzymes such as InhA [14], DNA gyrase [15], and MmpL3 [16,17].

Despite the growing interest in this class of compounds, only a limited number of reviews have specifically focused on thiazolidin-4-one derivatives with antitubercular activity [13]. Therefore, this review aims to provide a comprehensive overview of recent advances (from 2021 to the present) in the design, synthesis, and biological evaluation of thiazolidin-4-one derivatives as potential antitubercular agents. Particular emphasis is placed on structure–activity relationship (SAR), mechanisms of action, and molecular docking studies, to identify key structural features that contribute to enhanced antimycobacterial efficacy and inform future drug development strategies.

## 2. Methodology

Among the numerous reviews on the activity of thiazolidin-4-ones [18,19,20,21,22], the scientific literature contains only one that strictly addresses their antitubercular activity [13]. For the present study, literature was selected from the period 2021 to the present, sourced from the following scientific databases: Scopus (Elsevier), SciFinder (Chemical Abstracts), and PubMed. Research articles, short communications, and reports were included in our analysis. The search was conducted using specific keywords, including “thiazolidin-4-one”, “4-thiazolidinone”, “thiazolidine-2,4-dione”, “2,4-thiazolidinedione”, “thiazolidinedione-2,4”, “2-thioxothiazolidin-4-one”, “rhodanine”, “2-iminothiazolidin-4-one”, and “pseudothiohydantoin”, along with terms related to antitubercular activity, such as “antimycobacterial activity”, “antitubercular activity”, and “antituberculosis activity”. The review focused solely on thiazolidin-4-one derivatives, excluding other structural analogs or isomers such as thiorhodanine, isorhodanine, and thiohydantoin.

## 3. Main Pathways for Synthesis of Thiazolidin-4-one Ring

**Figure 1:**
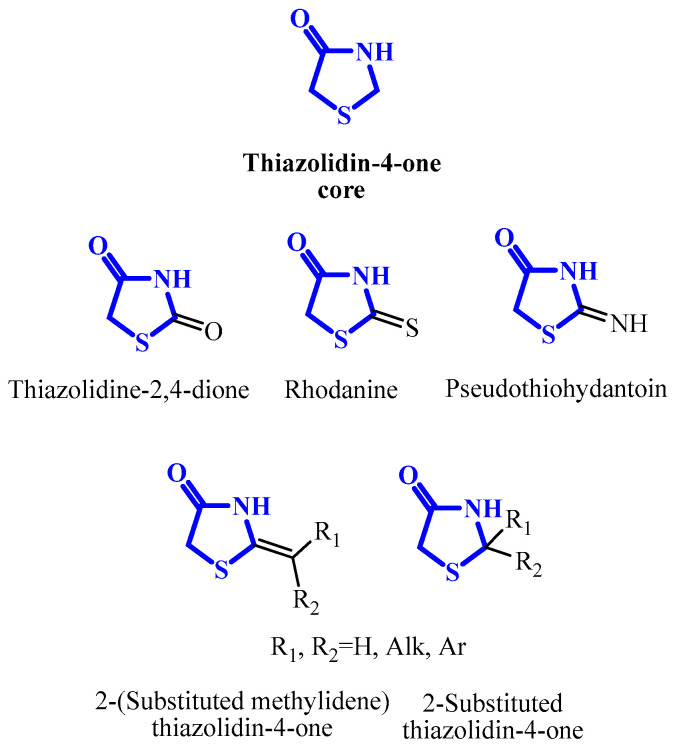
Different thiazolidin-4-one derivatives.

The compounds with thiazolidin-4-one core (colored blue in Figure 1) include derivatives of thiazolidine-2,4-dione (TZD), 2-thioxothiazolidin-4-one (rhodanine), 2-iminothiazolidin-4-one (pseudothiohydantoin), and 2-alkyl/arylthiazolidin-4-one.

**Scheme 1:**
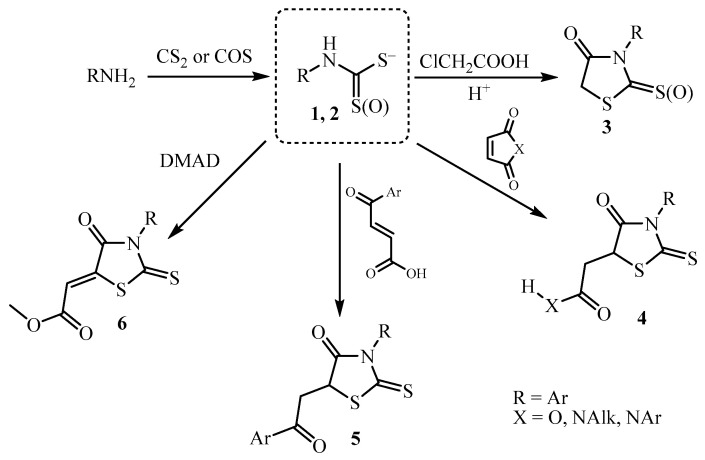
Synthesis of rhodanine and thiazolidine-2,4-dione rings using dithiocarbamates and thiocarbamates.

One of the classical approaches for the synthesis of thiazolidin-4-one ring involves a [2+3]-cyclocondensation reaction. In this process, various derivatives such as 2-halogencarboxylic acids, maleic acid derivatives, aroylacrylic acids, and dimethyl acetylenedicarboxylate (DMAD) are used as equivalents of the dielectrophilic synthon [C_2_]^2+^. These compounds react with N,S-nucleophiles, including dithiocarbamates (**1**) and thiocarbamates (**2**), leading to the formation of 3-substituted rhodanine or thiazolidine-2,4-dione (**3**) [23,24,25,26], 3-substituted rhodanine-5-acetamide derivatives (**4**) [27], 3-substituted 5-aroylmethylrhodanine (**5**) [28], or 3-substituted derivatives of (4-oxothiazolidin-5-ylidene)acetic acid (**6**) [29] (Scheme 1).

**Scheme 2:**
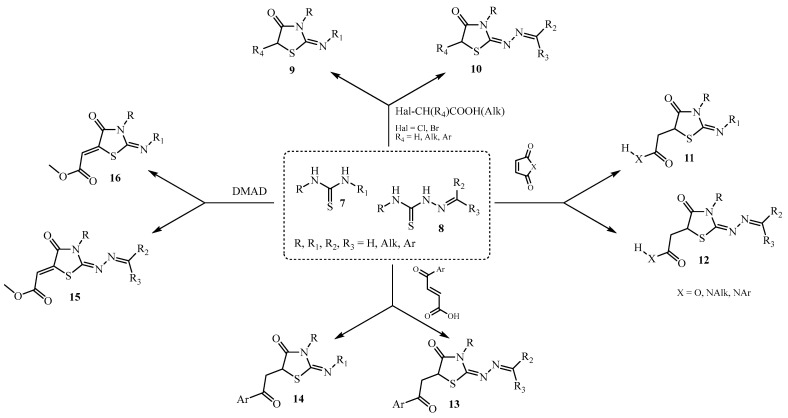
Synthesis of pseudothiohydantoin ring using thioureas and thiosemicarbazones.

The same dielectrophilic synthon [C_2_]^2+^, which includes 2-halogencarboxylic acids, maleic acid derivatives, aroylacrylic acids, and DMAD, along with other N,S-nucleophiles such as thioureas and thiosemicarbazones, is utilized for the synthesis of pseudothiohydantoin (2-iminothiazolidin-4-one) derivatives. The products of these reactions include 2-iminothiazolidin-4-ones (**9**, **10**) [30,31,32,33], (2-imino-4-oxothiazolidin-5-yl)acetic acid derivatives (**11**, **12**) [11,12,34], 5-aroylmethyl-2-iminothiazolidin-4-ones (**13**, **14**) [11], and (2-imino-4-oxothiazolidin-5-ylidene)acetic acid derivatives (**15**, **16**) [12,35] which are presented in Scheme 2.

**Scheme 3:**
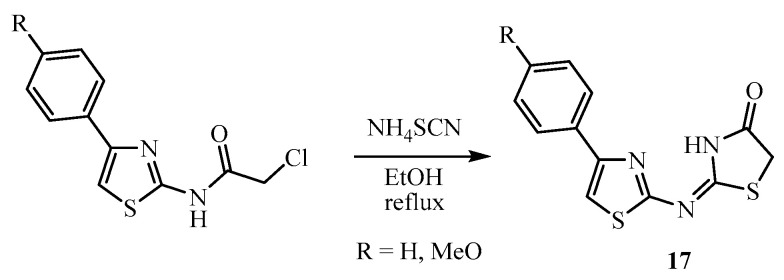
Synthesis of 2-iminothiazolidin-4-one derivatives.

Another method of constructing the 2-minothiazolidin-4-one is the cyclization of 2-chloroacetamide derivatives with ammonium thiocyanate in ethanol. The result of this reaction is the formation of 2-iminothiazolidin-4-one compounds (**17**) with a good yield of 40–50% (Scheme 3) [36].

**Scheme 4:**
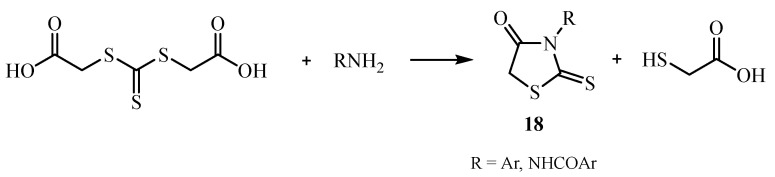
Synthesis of rhodanine derivatives.

A convenient and efficient method for the synthesis of 3-substituted rhodanines (**18**), particularly from amines or hydrazides of aromatic carboxylic acids, is the Holmberg method. This approach involves the reaction of thiocarbonyl-bis-thioglycolic acid with amino derivatives or hydrazides in an ethanol or ethanol–water medium [37,38] (Scheme 4).

**Scheme 5:**
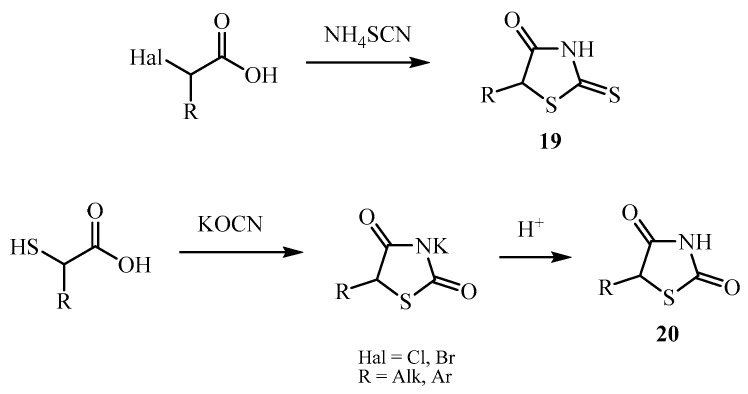
Synthesis of 3-unsubstituted TZD and rhodamine derivatives.

3-Unsubstituted rhodanine derivatives (**19**) can be obtained by the reaction of 2-halogencarboxylic acids and ammonium or alkali metal thiocyanates [39,40], whereas 3-unsubstituted thiazolidine-2,4-diones (**20**) are synthesized by the reaction of thioglycolic acid with potassium cyanate [41] (Scheme 5).

**Scheme 6:**
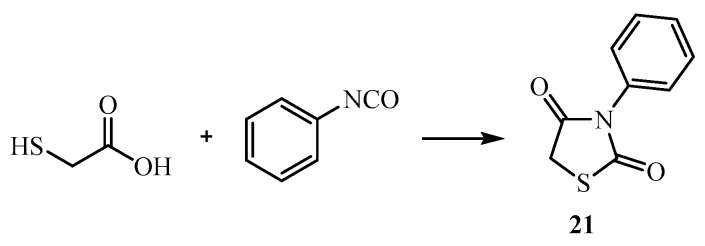
Synthesis of 3-substituted TZD.

On the other hand, 3-substituted thiazolidine-2,4-diones (**21**) are obtained following the reaction of thioglycolic acid with aryl isocyanates [42,43] (Scheme 6).

**Scheme 7:**
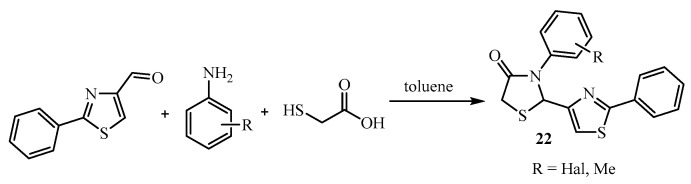
Synthesis of 2-heteroarylthiazolidin-4-ones.

Thioglycolic acid can also be used for the synthesis of 2-aryl/heteroarylthiazolidin-4-ones in a three-component reaction. The condensation of substituted aromatic amines in toluene, followed by cyclocondensation with thioglycolic acid, leads to the formation of 2-heteroarylthiazolidin-4-ones (**22**) [44] (Scheme 7).

**Scheme 8:**
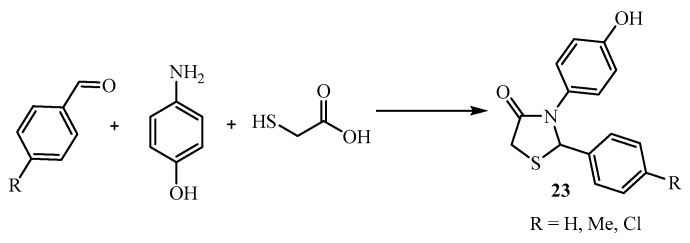
Synthesis of 2-arylthiazolidin-4-ones.

Another example of a three-component reaction is the synthesis of 2,3-diarylthiazolidin-4-ones (**23**) using *N,N*’-dicyclohexylcarbodiimide (DCC) in tetrahydrofuran (THF) as the solvent [45] (Scheme 8).

**Scheme 9:**
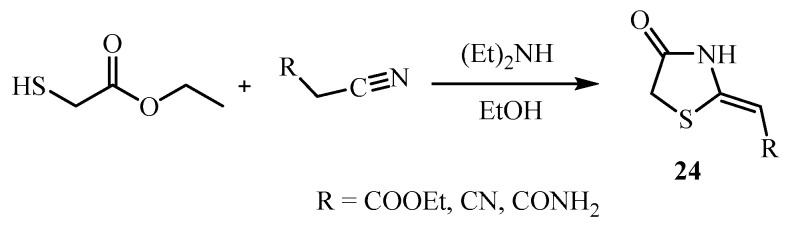
Synthesis of 2-methylidenethiazolidin-4-ones.

The esters of thioglycolic acid have been used for the synthesis of 2-methylidenethiazolidin-4-ones. Based-catalyst condensation of ethyl thioglycolate with methylene active nitriles such as ethyl 2-cyanoacetate, malononitrile, or 2-cyanoacetamide has led to obtaining 2-methylidenethiazolidin-4-ones (**24**) [46]. Scheme 9 illustrates the synthesis process.

The acid hydrolysis method for pseudohydantoins was first proposed by Volhard in 1874 [47] and has since found broad practical application in the synthesis of the TZD ring [48,49].

**Scheme 10:**
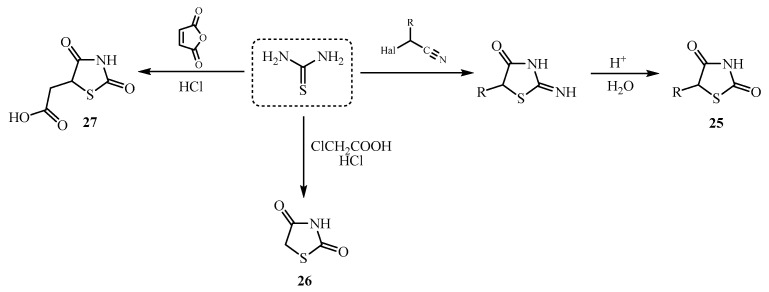
Synthesis of TZD ring by the reaction of cyclocondensation with following acid hydrolysis.

The reaction of [2+3]-cyclocondensation using thioureas as N,S-nucleophiles with following acid hydrolysis is a traditional approach to the synthesis of TZDs (**25**–**27**) (Scheme 10). In these reactions, 2-halogenonitriles [50], chloroacetic acid [51], or maleic anhydride [52] have been used as equivalents of the dielectrophilic synthon [C_2_]_2_^+^.

**Scheme 11:**
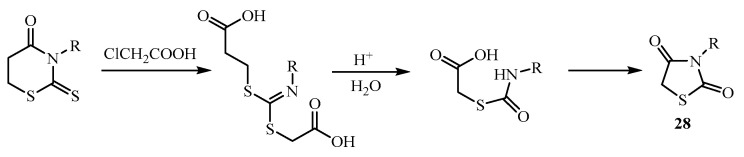
Synthesis of thiazolidine-2,4-diones via ring transformation reaction.

An alternative approach to the synthesis of TZD involves ring transformation reactions. In this process, 2-thioxo-1,3-thiazanones undergo ring transformation to yield TZD (**28**) (Scheme 11) [53].

The above-mentioned synthetic approaches presented in this section are classical strategies for constructing the thiazolidin-4-one system and remain relevant in modern applications [54,55,56,57,58].

## 4. Structure–Activity Relationship (SAR)

### 4.1. 2-Alkyl/Arylthiazolidin-4-ones

**Figure 2:**
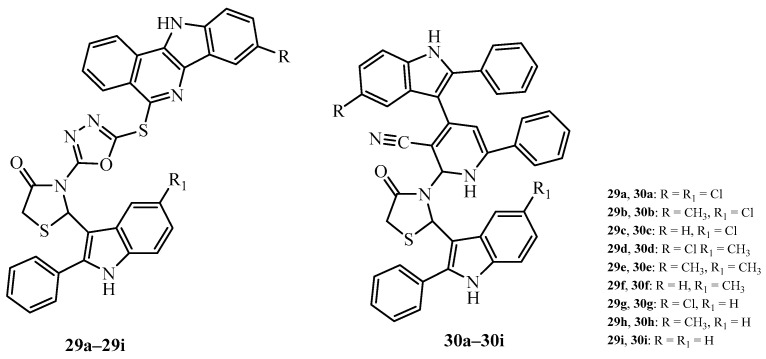
The structures of 3-substituted 2-phenylindol-3-ylthiazolidin-4-one derivatives **29a**–**29i** and **30a**–**30i**.

Verma and Saundane synthesized a series of 2-phenylindol-3-ylthiazolidin-4-one derivatives bearing a 1,3,4-oxadiazole moiety and evaluated their antitubercular activity against *M. tuberculosis* H_37_Rv [59]. Compounds **29a**–**29i** (Figure 2) showed antitubercular activity at MIC values in the range of 1.5–>100 µg/mL. The most effective compound was derivative **29d** (MIC = 1.5 µg/mL). As a continuation of this research, Verma et al. modified 2-phenylindol-3-ylthiazolidin-4-one derivatives by the indolyl-pyridine moiety in the third position of the thiazolidine ring [60]. This modification led to obtaining compounds **30a**–**30i** and improvement in antitubercular activity compared to series compounds **29a**–**29i** (MIC = 0.8–25 µg/mL). The most effective compounds (**30e**, **30h**, and **30i**) among derivatives **30** were 32-fold more active than their analogs **29** (MIC 0.8 vs. 25 µg/mL), whereas the analog of **29d,** compound **30d,** showed antitubercular activity at the same concentration (MIC = 1.5 µg/mL).

**Figure 3:**
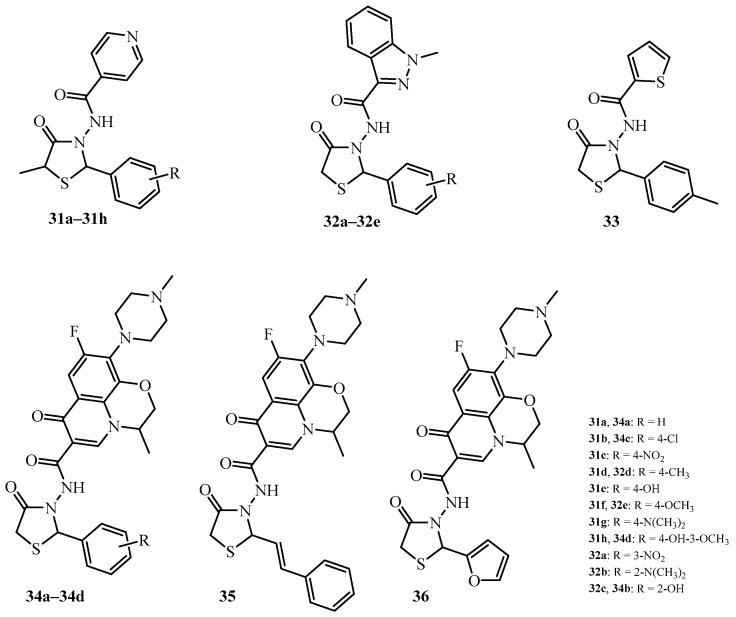
The structures of 3-acylamino-2-arylthiazolidin-4-ones.

Alghamdi et al. synthesized a series of 2-arylthiazolidin-4-one hybrids (**31a**–**31h**) with an isonicotinoylamino substituent at the third position of the thiazolidine ring (Figure 3). These derivatives showed weak antituberculosis activity against the *Mtb* H_37_Rv strain with MICs in the range of 25–100 µg/mL. The most active among them was compound **31h** (MIC = 25 µg/mL) with 4-hydroxy-3-methoxyphenyl substituent in the second position [61]. The replacement of isonicotinoylamino substituent by the 1-methylindazole-3-carboxamide (compounds **32a**–**32e**) led to slightly improved antimycobacterial activity against *Mtb* H_37_Rv (MIC = 13.79–>100 µg/mL) [62], wherein the most active compound **32a** (MIC = 13.79 µg/mL) contained 3-nitrophenyl substituent in the second position of thiazolidine ring. The worst activity against *Mtb* showed compound **32d** with 4-tolyl substituent in position 2 (MIC > 100 µg/mL). The same is true for the derivative **31d** (MIC = 100 µg/mL), as well as for compound **33**, which also has a 4-tolyl substituent in position 2 and exhibited only 38% growth inhibition of *Mtb* at a concentration of 12.5 µg/mL [54].

Chintakunta and Subbareddy synthesized ofloxacin containing thiazolidin-4-one derivatives (**34a**–**34d**, **35**, and **36**) and investigated their antitubercular activity [63] (Figure 3). The introduction ofloxacin moiety in the third position of the thiazolidine ring significantly improved antitubercular activity. The MICs for compounds **34b**–**34d**, **35**, and **36** were compared to those for isoniazid and ethambutol (MIC = 1.6 µg/mL) and showed two-fold better than activity pyrazinamide (MIC 1.6 vs. 3.125 µg/mL).

**Figure 4:**
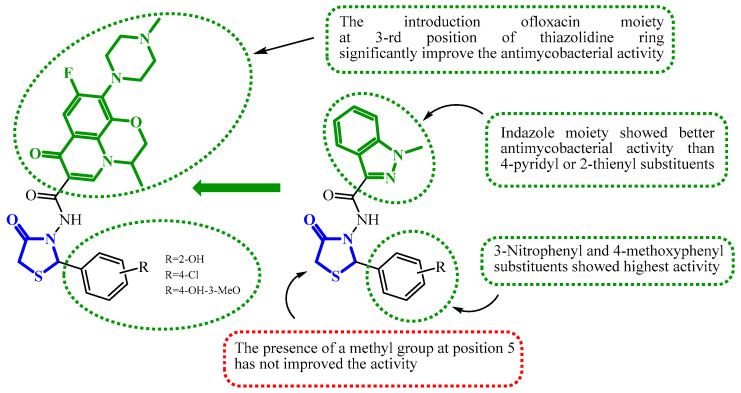
The SAR for 3-acylaminothiazolidin-4-ones.

The influence of structural fragments on the antitubercular activity of 3-acylaminothiazolidin-4-ones is depicted in Figure 4.

**Figure 5:**
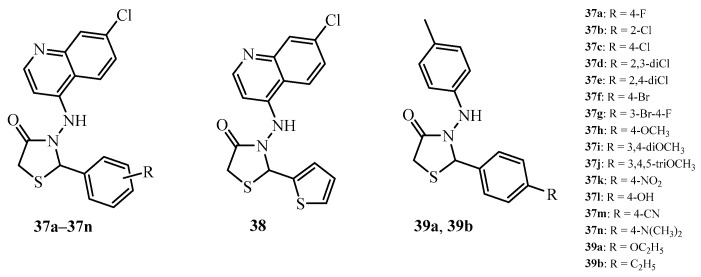
The structures of 3-arylaminothiazolidin-4-one derivatives.

Salve et al. designed and synthesized of new 3-[(7-chloroquinolin-4-yl)amino]thiazolidine-4-ones (**37a**–**37n**, **38**) [64]. A series of these derivatives (Figure 5) displayed good antimycobacterial activity in the range of MICs from 1.56 to 12.5 µM. Derivatives **37f**, **37h**, and **37i**, containing 4-bromophenyl, 4-methoxyphenyl, and 3,4-dimethoxyphenyl substituents at position 2, exhibited antimycobacterial activity comparable to the reference drugs pyrazinamide and ciprofloxacin (MIC = 3.12 µM). On the other hand, compounds with 2,4-dichlorophenyl, 3-bromo-4-fluorophenyl, 4-dimethylaminophenyl, and 2-thienyl substituents at position 2 of the thiazolidine ring (compounds **37e**, **37g**, **37n**, and **38**) demonstrated activity superior to pyrazinamide or ciprofloxacin and comparable to ethambutol (MIC = 1.56 µM). The remaining derivatives of this series were active at concentrations of 6.25–12.5 µM [64].

The replacement of the 3-(7-chloroquinolin-4-yl)amino substituent with the 4-tolylamino group (**39a**, **39b**) decreased the antimycobacterial activity against *Mtb* H_37_Rv (MIC = 12.5 µg/mL) [65].

**Figure 6:**
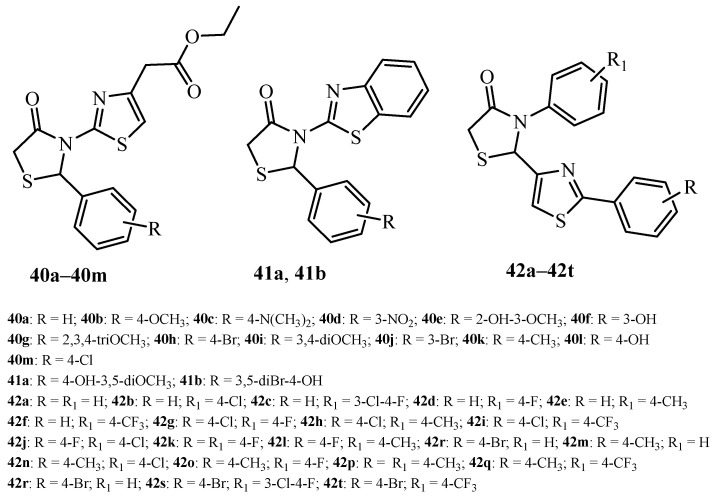
Thiazolidin-4-ones with 1,3-thiazole moiety.

Othman and colleagues designed and synthesized new thiazolidin-4-one conjugates with 1,3-thiazole at position 3 of the thiazolidine ring (**40a**–**40m**) (Figure 6). The obtained compounds were evaluated for their antitubercular activity against the *Mtb* H_37_Ra (drug-sensitive strain), MDR, and XDR *Mtb* strains. All the tested compounds displayed potent to mild antitubercular activity with MIC in the 0.12–62.5 µg/mL range against drug-sensitive strains. The compound **40h** with 4-bromophenyl substituent showed activity against *Mtb* H_37_Ra comparable to the reference drug isoniazid (MIC = 0.12 µg/mL). Worth noticing that compounds **40b**, **40m** (MIC = 0.48 µg/mL), and **40i** (MIC = 0.98 µg/mL) also showed good antitubercular potential. Other derivatives exhibited moderate or mild activity with MIC range of 1.95–7.81 µg/mL and 15.63–62.5 µg/mL, respectively [55].

Additionally, compound **40h** showed the highest activity to the MDR and XDR strains with MIC = 1.95 and 7.81 µg/mL, correspondingly. Also, high effectiveness was shown by 4-methoxy or 4-chloro analogs of **40h**, compounds **40b** and **40m**. Their MICs against the MDR *Mtb* strain were 3.9 and 7.81 µg/mL, respectively. Other compounds displayed a moderate effect with MIC = 15.63–31.25 µg/mL (**40e**, **40i**, and **40j**), mild activity in the range of concentration 62.5–125 µg/mL (**40a**, **40e**, **40f**, **40g**, **40k**, and **40l**), or were inactive (**40a**, **40c**, **40d**, **40f**, **40g**, and **40j**–**40l**) towards both the MDR and XDR *Mtb* strains. Details of the results are presented in Table 1.

The introduction of the benzothiazole moiety at position 3 of the thiazolidine ring generally resulted in decreased antitubercular activity (Figure 6). The activity of compounds **41a** and **41b**, which were the most effective against *Mtb* H_37_Rv in this series, had an MIC of 6.25 µg/mL. This concentration was comparable to that of the reference antibiotic streptomycin [66].

The replacement of substituents (2-aryl and 3-thiazolyl) at positions 2 and 3 of derivatives **40a**–**40m** led to a decrease in the antitubercular activity of compounds **42a**–**42t** against *Mtb* H_37_Ra (MIC_90_ = 61.25–>100 µM). Nevertheless, some of these compounds (**42j**, **42o**, and **42t**) showed activity against *Mycobacterium bovis* BCG with MIC_90_ values of 5.44, 4.43, and 5.10 µM, respectively [44].

**Figure 7:**
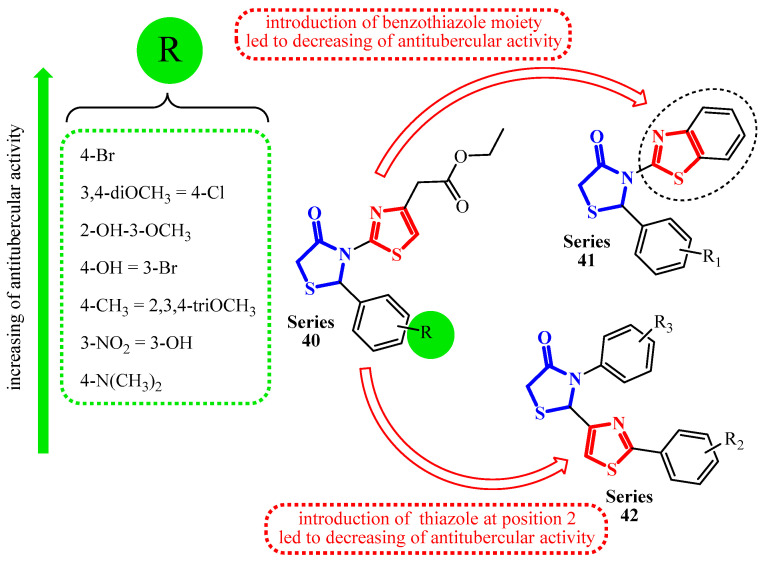
The structure–activity relationship of thiazolidin-4-ones with a 1,3-thiazole moiety.

The main trends in structure–activity relationships for thiazolidin-4-ones containing a 1,3-thiazole moiety are presented in Figure 7. Figure 7 illustrates the influence of changing the position of the thiazole moiety (highlighted in red) within the thiazolidin-4-one ring (shown in blue).

**Figure 8:**
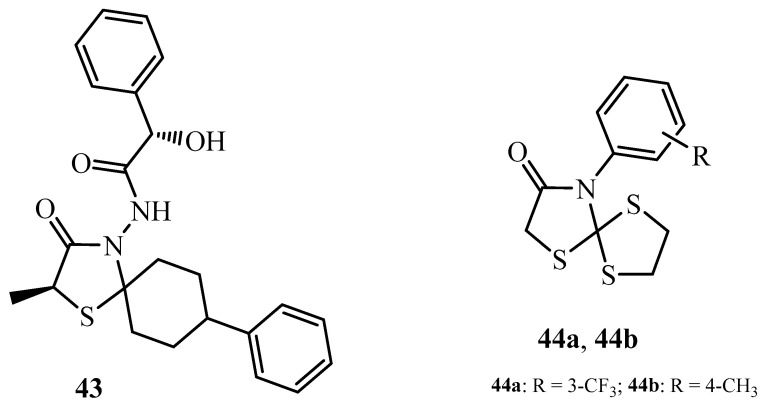
The structures of spirothiazolidin-4-one derivatives.

Trawally et al. obtained a series of spirothiazolidin-4-one derivatives based on mandelic acid hydrazide and evaluated them in vitro for their antitubercular activity. Only compound **43** (Figure 8) displayed 98% inhibition at a concentration of 6.25 µg/mL, which suggests a moderate antitubercular effect against *Mtb* H_37_Rv compared to the reference drug rifampicin (MIC = 0.125 µg/mL) [67].

Other spirothiazolidin-4-one derivatives described by Pandey et al. were tested for activity against *Mtb* H_37_Rv. The results reveal that only two compounds (**44a** and **44b**) showed antitubercular activity with an MIC of 6.25 µg/mL, which is better than the rest of the compounds in the series (MIC > 12.5 µg/mL) [68].

It is worth noting that Moulishankar and Thirugnanasambandam developed a quantitative structure–activity relationship (QSAR) model for certain 2-arylthiazolidin-4-one derivatives, which are potent antitubercular agents. This research investigates the contribution of molecular descriptors of thiazolidin-4-ones to their antitubercular activity. The QSAR equation identifies the molecular descriptors that are either positively or negatively correlated with antitubercular activity. Additionally, it was found that the polarizability, electronegativities, surface area contributions, and presence of halogen atoms in the 2-arylthiazolidin-4-one derivatives increase, thereby enhancing their antitubercular activity [69].

### 4.2. 2-Iminothiazolidin-4-ones (Pseudothiohydantoins)

**Figure 9:**
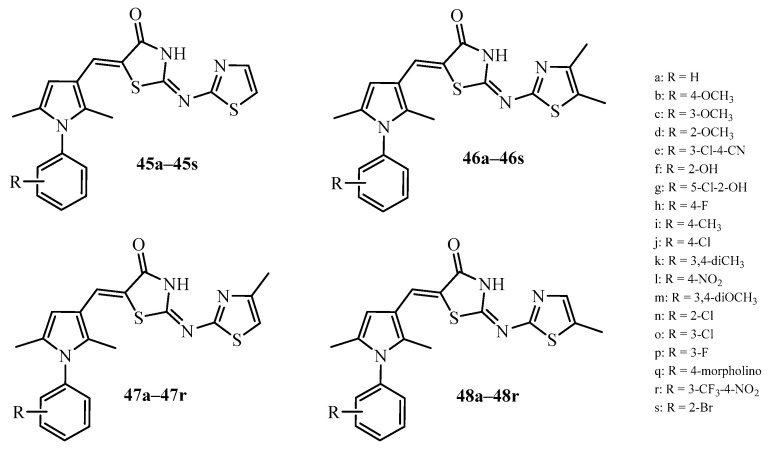
The structures of pyrrole-thiazolidin-4-one hybrids with a thiazole moiety.

Ahmed and co-workers synthesized a series of pyrrole-thiazolidin-4-one hybrids (compounds **45**, **46**, **47**, and **48**) as promising antitubercular agents (Figure 9) [56,70]. All these derivatives contain a thiazole moiety in their structure. Evaluation of antitubercular activity against the *Mtb* H_37_Rv strain showed that compounds **45a**–**45s** with unsubstituted thiazole moieties were generally more active than derivatives **46a**–**46s** with a 4,5-dimethylthiazole moiety. The compound **45k** with 3,4-dimethylphenyl substituent was the most active among all the tested hybrids (MIC = 0.5 µg/mL). Moreover, hybrid **45k** was two-fold better than the reference drugs streptomycin and ethambutol with MIC = 1 µg/mL. But, its analog **46k** showed 8-fold worse activity (MIC = 4 µg/mL). However, it is worth noting that compounds **45d**, **45h**, **45q**, **46c**, **46n**, **46o**, and **46s** displayed good antitubercular activity with MICs in the range of 2–4 µg/mL. Other derivatives of series **45** and **46** showed moderate or mild antitubercular effect (MIC = 8–64 µg/mL), or they were also inactive (MIC > 64 µg/mL). Moreover, none of the tested derivatives were active against other mycobacterial strains (*Mycobacterium abscessus*, *Mycobacterium fortuitum*, and *Mycobacterium chelonae*). Their MICs were >64 µg/mL [56].

The compounds from series **47** and **48**, containing 4-methylthiazole or 5-methylthiazole moieties, demonstrated a level of antitubercular activity comparable to that of series **45** and **46**. The compounds **48a**–**48r** with 5-methylthiazole were more active than the analogs of series **47** with a 4-methylthiazole moiety. The most active compounds among the tested derivatives were **48a** and **48k**, bearing phenyl and 3,4-dimethylphenyl substituents, respectively (Figure 9). They exhibited excellent activity with MIC = 0.5 µg/mL. The good antitubercular activity also showed derivatives **48h**–**48j** and **48o** with MIC values of 4 µg/mL. Other compounds from series **47** and **48** exhibited moderate to mild activity or were inactive, with MIC values ranging from 8 to 64 µg/mL or exceeding 64 µg/mL. Like the compounds of series **45** and **46**, hybrids **47** and **48** were inactive against *M. abscessus*, *M. fortuitum*, and *M. chelonae* strains [70].

**Figure 10:**
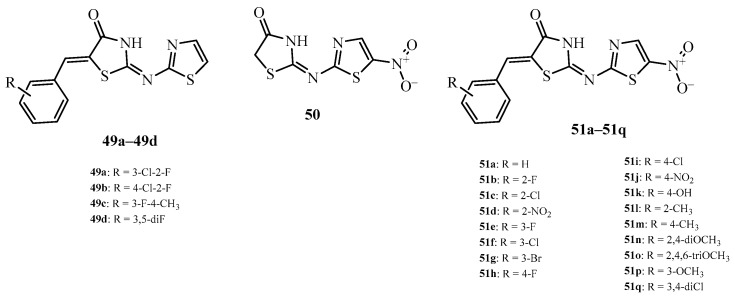
The structures of 5-arylidenethiazolidin-4-one derivatives with thiazole and 5-nitrothiazole moieties.

As reported by Kumar et al., the thiazolidin-4-one-based derivatives **49a**–**49d** (Figure 10), containing a thiazole moiety, exhibited activity against *Mycobacterium tuberculosis* H_37_Rv comparable to the reference drug streptomycin (MIC = 6.25 µg/mL). In the case of compound **49a** (MIC = 0.78 µg/mL), this activity was 4-fold and 8-fold greater than that of the reference drugs pyrazinamide and streptomycin, respectively [71].

The introduction of a 5-nitro group into the thiazole ring significantly improved the antitubercular activity compared to the unsubstituted thiazole analogs (Figure 10). The compounds **51a**–**51q**, except for **50** (MIC_90_ = 31.25 µM), exhibited antitubercular activity with MIC_90_ values ranging from <0.24 to 2 µM. The most effective compounds were **51c**, **51g**, **51h**, **51i**, **51l**, **51n**, and **51q** with MIC_90_ values less than 0.24 µM. Additionally, all the tested compounds were non-cytotoxic against HEK-293 cells at a concentration of 32 µg/mL (CC_50_ > 32 µg/mL) [72].

**Figure 11:**
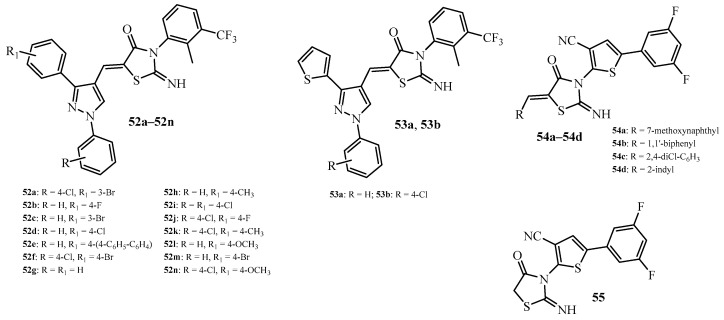
The structures of 3,5-disubstituted 2-iminothiazolidin-4-ones.

Kamat et al. described the synthesis and antimycobacterial activity of 2-iminothiazolidin-4-one derivatives bearing a pyrazole moiety (**52a**–**52n**, **53a**, and **53b**) (Figure 11). The compounds were tested against replicating and nonreplicating *M. tuberculosis* H_37_Rv strains and exhibited activity with MIC values ranging from 3.03 to >100 µg/mL. Five derivatives—**52a**, **52c**, **52d**, **52e**, and **52f**—showed promising potential as antitubercular agents against replicating *Mtb* H_37_Rv (MIC = 3.03–22.55 µg/mL). Compound **52a** with 4-chlorophenyl substituent demonstrated the highest potential within the series (MIC = 3.03 µg/mL). It is worth noting that the in vitro cytotoxicity study of the most effective compounds against Vero cells revealed a low toxicity level, with an IC_50_ ranging from 33.5 to 84.45 µg/mL [73].

As reported by Nayak, thiazolidin-4-one derivatives containing a thiophene moiety (**54a**–**54d**) inhibited *M. tuberculosis* H_37_Rv at concentrations ranging from 12.5 to 50 µg/mL. The absence of a substituent at position 5 (compound **55**) resulted in reduced antimycobacterial activity, with an inhibitory concentration of 100 µg/mL [74].

**Figure 12:**
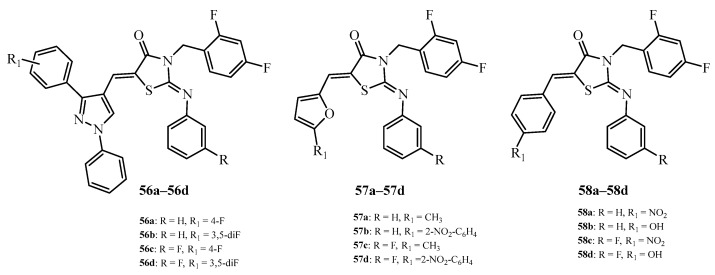
The structures of 2,3,5-trisubstituted 2-iminothiazolidin-4-one derivatives.

The modification of the 2-imino group of the thiazolidine ring with phenyl or 3-fluorophenyl substituents significantly enhanced antitubercular activity. The MIC values against *M. tuberculosis* H_37_Rv strain for the tested compounds (**56a**–**56d**, **57a**–**57d**, and **58a**–**58d**) ranged from 1.6 to 6.25 µg/mL (Figure 12). The majority of the compounds demonstrated excellent activity, with MIC values of 1.6 µg/mL, which were comparable to the reference drug isoniazid and superior to pyrazinamide and ethambutol (MIC = 3.12 µg/mL) [75].

**Figure 13:**
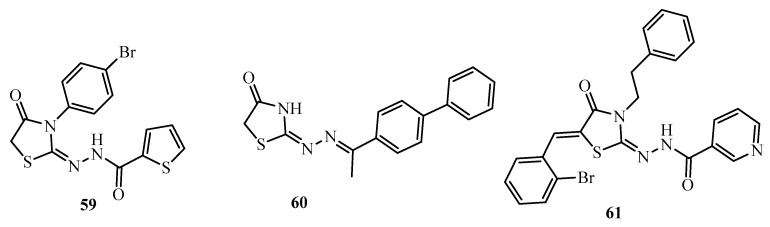
The structures of 2-hydrazinylidenethiazolidin-4-ones.

Among the 2-hydrazinylidenethiazolidin-4-ones tested against *M. tuberculosis* H_37_Rv, only compounds **59**, **60**, and **61** exhibited moderate antitubercular activity (Figure 13). Compound **59** demonstrated 76% inhibition of *M. tuberculosis* at a concentration of 12.5 µg/mL [54]. Derivative **60** showed antitubercular activity with an MIC value of 12.5 µg/mL [76]. A slightly better activity was observed for derivative **61**, which inhibited the growth of *M. tuberculosis* H_37_Rv with a minimum inhibitory concentration of 6.25 µg/mL (12.32 µM) [57].

**Figure 14:**
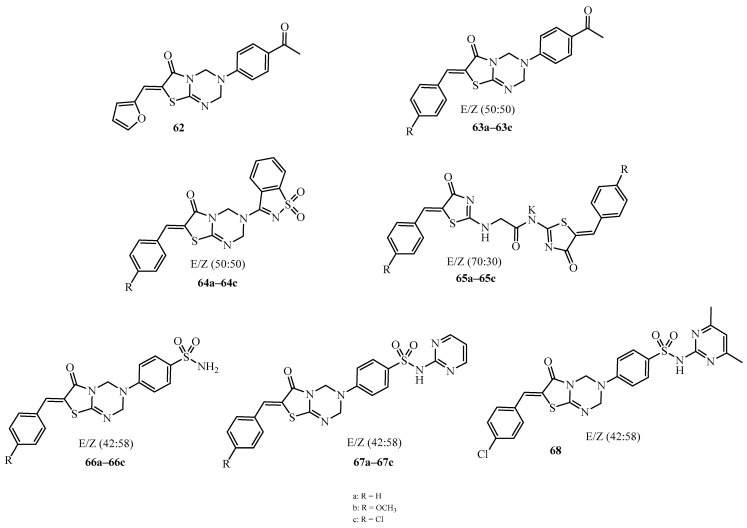
The structures of thiazolo [3,2-*a*][1,3,5]triazine derivatives (**62**, **63a**–**63c**, **64a**–**64c**, **66a**–**66c**, **67a**–**67c**, and **68**) and bis-thiazolidin-4-ones (**65a**–**65c**).

Younis and colleagues designed, synthesized, and evaluated the antimycobacterial activity of novel thiazolo [3,2-*a*][1,3,5]triazine (**62**, **63a**–**63c**, **64a**–**64c**, **66a–66c**, **67a**–**67c**, and **68**) and bis-thiazolidin-4-one (**65a**–**65c**) derivatives (Figure 14). To assess their antitubercular effects, the synthesized compounds were tested against the drug-sensitive *Mtb* H_37_Ra strain, as well as multidrug-resistant (MDR) and extensively drug-resistant (XDR) strains [77]. Among the tested compounds, derivatives **62** and **64c** exhibited the highest efficacy. These compounds were active against both drug-sensitive and drug-resistant strains. Their MIC values were 2.49 µM (**62**) and 2.28 µM (**64c**) against the *Mtb* H_37_Ra strain; 9.91 µM (**62**) and 18.14 µM (**64c**) against the MDR strain; and 39.72 µM (**62**) and 36.31 µM (**64c**) against the XDR strain. Replacing the furan-2-ylmethylidene substituent in compound **62** with benzylidene groups in compounds **63a**–**63c** resulted in a significant decrease in the antimycobacterial activity (MIC 2.49 µM vs. 60.4–85.99 µM). It is also worth noting that compounds **65b** and **66a** demonstrated good activity against both drug-sensitive and drug-resistant strains. The MIC values for compound **65b** were 3.60 µM (*Mtb* H_37_Ra), 28.89 µM (MDR), and 115.52 µM (XDR), while for compound **66a**, they were 4.43 µM, 17.73 µM, and 70.94 µM, respectively. Moreover, compounds **64b**, **66b**, and **68** exhibited antimycobacterial activity against both the *Mtb* H_37_Ra and MDR strains, with MIC values ranging from 8.97 to 17.55 µM and 39.03 to 71.85 µM, respectively. The remaining derivatives showed moderate to mild activity, or no antimycobacterial effect [77].

### 4.3. Thiazolidine-2,4-diones

**Figure 15:**
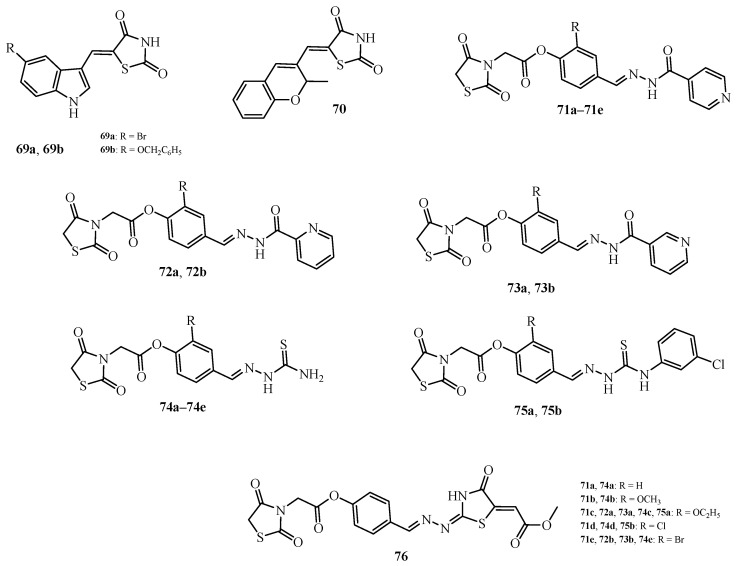
The structures of 3 and 5-substituted TZD.

Angelova et al. described the synthesis of novel TZD derivatives substituted in position 5. In this work, the authors replace the chromene scaffold (compound **70**) with the indole heterocycle (compounds **69a**, **69b**) to test the possibility of increasing the antimycobacterial activity (Figure 15). The results of antimycobacterial tests confirmed the potential of the new compounds as antitubercular agents. Compounds **69a** and **69b** inhibited *M. tuberculosis* H_37_Rv with MIC values of 1.55 µM and 1.43 µM, respectively. These values were lower than those of the reference drugs isoniazid (MIC = 1.82 µM) and ethambutol (MIC = 2.00 µM) [78]. However, the results were not superior to those obtained for the previously described chromene derivative **70** (MIC = 0.36 µM) [79]. It is worth emphasizing that compounds **69a** and **69b** exhibited low cytotoxicity against HEK-293 cells (IC_50_ > 200 µM), thereby demonstrating a high selectivity index (SI > 129).

Our study presents the design, synthesis, antitubercular activity evaluation, and drug interaction analysis of novel TZD-based compounds containing pyridinecarbohydrazide (**71a**–**71e**, **72a**, **72b**, **73a**, and **73b**) and thiosemicarbazone moieties (**74a**–**74e**, **75a**, and **75b**) (Figure 15) [80]. The results of our study revealed that TZD–pyridine-4-carbohydrazide hybrids (**71a** and **71c**–**71e**) exhibited excellent activity against *M. tuberculosis* H_37_Rv, with MIC values ranging from 0.078 to 0.283 µM. Furthermore, all of these compounds (**71a** and **71c**–**71e**) demonstrated superior activity compared to the reference drugs ethambutol (MIC = 2.45 µM) and streptomycin (MIC = 0.43 µM). Notably, compounds **71a** (MIC = 0.078 µM) and **71d** (MIC = 0.144 µM) outperformed isoniazid (MIC = 0.228 µM) and rifampicin (MIC = 0.152 µM) in terms of inhibitory potency against the *Mtb* H_37_Rv strain. TZD–pyridine-4-carbohydrazide hybrids (**71a** and **71c**–**71e**) also exhibited notable activity against the *Mtb*-MDR 210 strain, with MIC values in the range of 8.38–18.08 µM.

The other series of TZD-based hybrids, namely TZD–thiosemicarbazones (**74a**–**74e**), exhibited good antimycobacterial activity against the *Mtb* H_37_Rv strain, with MIC values ranging from 0.63 to 9.27 µM. Within this series, compound **74c** was the most effective, with an MIC of 0.63 µM.

**Figure 16:**
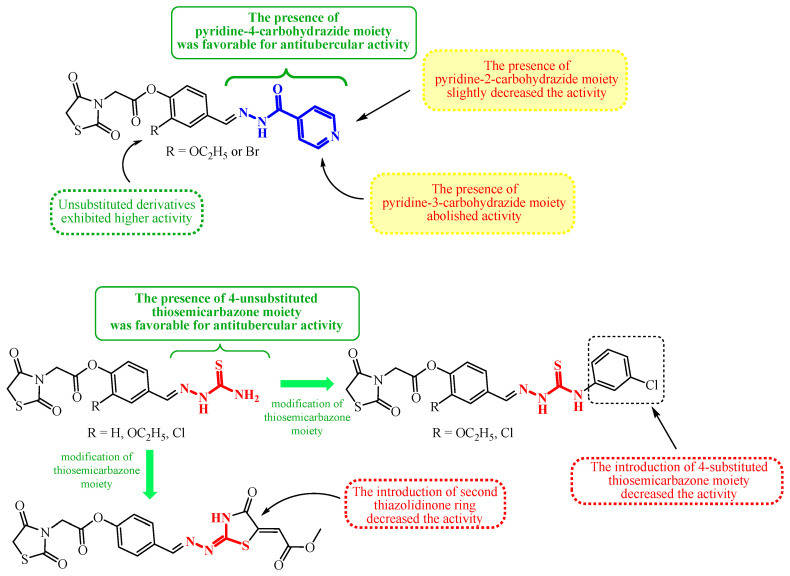
The SAR of 3-substituted TZD.

The substitution of the pyridine-4-carbohydrazide moiety with pyridine-2-carbohydrazide (compounds **72a** and **72b**) led to a moderate reduction in antitubercular activity (MICs 0.262–0.283 µM vs. 2.26–4.19 µM). In contrast, replacement with pyridine-3-carbohydrazide (**73a** and **73b**) resulted in a complete loss of activity (MICs 578.6 and 268.2 µM, respectively) (Figure 16). In a similar manner, trends were observed for the TZD–thiosemicarbazones (**74c** and **74d**). The introduction of a 4-substituted thiosemicarbazone moiety (**75a** and **75b**) led to a marked reduction in activity against the *Mtb* H_37_Rv strain (MICs 0.63 vs. 31.56 µM and 5.17 vs. 32.17 µM). The incorporation of a second thiazolidinone ring into molecule **74a**, yielding compound **76**, resulted in a significant reduction in activity, with MIC increasing from 2.84 µM to 34.6 µM (Figure 16).

Moreover, based on the fractional inhibitory concentration index (FICI), the potency of two-drug combinations of the new compounds with one of four reference drugs in combination therapy was evaluated. Some of the tested compounds (**71e** and **76**) showed a synergistic effect with isoniazid against the *Mtb* H_37_Rv strain (FICI = 0.474), while compounds **74c** and **74e** exhibited synergy with rifampicin against the *Mtb* 192 strain, with FICI values of 0.375 and 0.187, respectively. These findings suggest that the compounds may serve as effective scaffolds for the development of new co-adjuvants in tuberculosis therapy [80].

**Figure 17:**
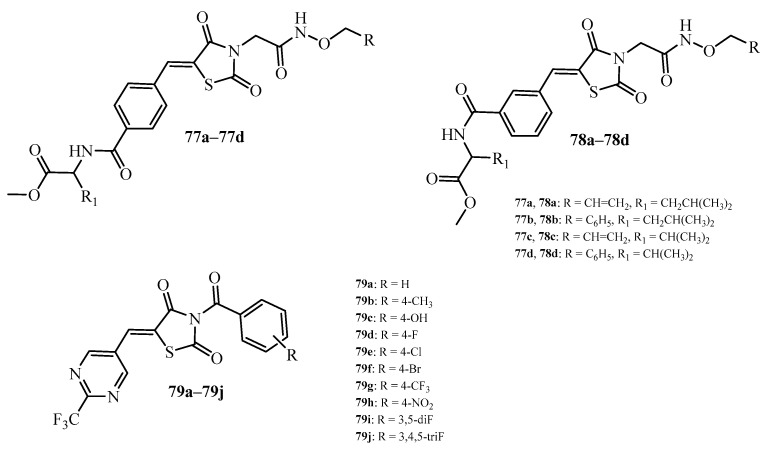
The structures of 3,5-disubstituted TZD.

TZD-hydroxamate derivatives (**77a**–**77d** and **78a**–**78d**) reported by Dak et al. demonstrated significant inhibition of intracellular *Mtb* H_37_Ra survival within RAW 264.7 macrophages (Figure 17). The most potent compound, **77d**, reduced bacterial survival by 83.2% after 72 h at a concentration of 128 µM. Other notably active derivatives included **77a** (67.3%), **77b** (80.3%), and **78b** (51.9%), all exhibiting over 50% inhibition. The observed structure–activity relationship indicated that activity was enhanced by *para*-substitution on the aromatic ring and the presence of an isopropyl group at the ester moiety attached to the benzene ring [81].

The series of pyrimidine-linked thiazolidinedione derivatives (**79a**–**79j**) exhibited good to excellent antimycobacterial activity, with MIC values ranging from 0.22 to 10.91 µM (Figure 17). The most promising antitubercular effects were observed for compounds **79g**, **79i**, and **79j**, which contained electron-withdrawing groups such as trifluoromethyl, 3,5-difluoro, and 3,4,5-trifluoro, respectively. Their MIC values were 0.22, 0.71, and 0.32 µM. Notably, compounds **79g** and **79j** showed greater potency than the reference drug isoniazid (MIC = 0.35 µM) [82].

### 4.4. 2-Thioxothiazolidin-4-ones (Rhodanines)

**Figure 18:**
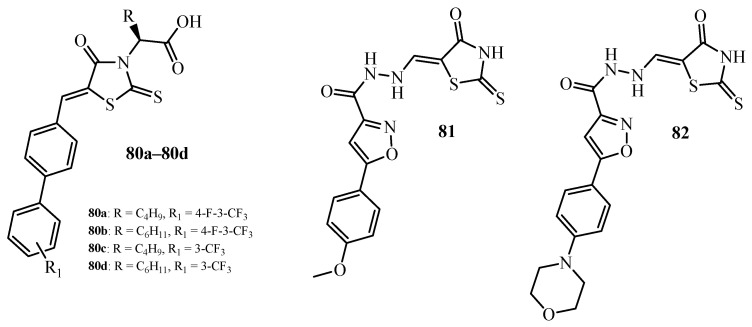
The structures of rhodanine derivatives with antitubercular effects.

Cheng and co-workers reported the antitubercular activity of 5-arylidenerhodanine-3-acetic acid derivatives (**80a**–**80d**) (Figure 18). These compounds exhibited mild activity against *Mtb* H_37_Rv, with MIC values ranging from 58.3 to 64.9 µM. Notably, compound **80a**, bearing a butyl substituent, exhibited better activity than its cyclohexyl analog **80b**, with MIC values of 58.3 µM and 63.8 µM, respectively. An opposite trend was observed for compounds **80c** and **80d**, with MIC values of 64.9 µM and 62.2 µM, respectively [58].

Other rhodanine derivatives linked to enamine-carbohydrazide, described by Maddipatla et al., generally exhibited moderate to mild antitubercular activity in vitro (MIC = 16–128 µg/mL). Only two derivatives, **81** and **82** (Figure 18), demonstrated good to excellent antitubercular activity, with MIC values of 8 and 1 µg/mL, respectively [83].

## 5. Mechanism of Action

In the process of discovering new drugs and mechanisms of action against *Mycobacterium tuberculosis*, the crystal structures of many key protein targets are extensively utilized. Among the most frequently studied are the following: enoyl-acyl carrier protein reductase (InhA), 3-oxoacyl-[acyl-carrier-protein] synthase 1 (KasA), (3R)-hydroxyacyl-ACP dehydratase heterodimer (HadAB), polyketide synthase (Pks13), fatty acid degradation protein D32 (FadD32), and the trehalose monomycolate transporter (MmpL3)—all of which play essential roles in the biosynthesis and transport of mycolic acids [84]. Additionally, common therapeutic targets include the following: decaprenylphosphoryl-ß-D-ribose 2′-oxidase (DprE1), which participates in the cell wall biogenesis [85,86]; mycobacterial zinc metalloprotease-1 (Zmp1), which is essential enzyme for intracellular survival and pathogenicity of *Mtb* [87]; or pantothenate synthetase (PS), which catalyzes the synthesis of pantothenate from D-pantoate and ß-alanine with the hydrolysis of ATP into AMP and inorganic pyrophosphate [88].

### 5.1. Enoyl-Acyl Carrier Protein Reductase (InhA)

According to Kamat et al., the most active compound among the series **52** (Figure 11), namely the 2-iminothiazolidin-4-one derivative bearing a pyrazole moiety with a *p*-chlorophenyl substituent (compound **52a**), was subjected to molecular docking to evaluate its potential binding modes. These modes were then analyzed and compared to those of the reference ligand, 5-{[4-(9*H*-fluoren-9-yl)piperazin-1-yl]carbonyl}-1*H*-indole, in complex with the enoyl-ACP reductase enzyme InhA (PDB ID: 00001p44). The docking score for compound **52a** was −6.41 kcal/mol, whereas the reference ligand exhibited a significantly higher binding affinity with a docking score of −12.05 kcal/mol. Critical amino acid residue analysis revealed a single C–H bond interaction between the trifluoromethyl group of compound **52a** and the Pro193 residue, with a bond length of 3.61 Å, as well as a π–π interaction with the Phe97 residue. Additionally, several π–alkyl interactions were observed between the substituted aryl rings of compound **52a** and surrounding amino acid residues [73].

Among the thiazolidine-4-one conjugates containing a thiazole moiety (**40a**–**40m**) (Figure 6), the most effective derivatives, **40b** and **40h**, exhibited notable inhibitory activity against the InhA enzyme, with IC_50_ values of 1.3 ± 0.61 µM and 1.06 ± 0.97 µM, respectively—comparable to that of isoniazid (IC_50_ = 0.23 ± 1.2 µM). Moreover, molecular docking studies demonstrated that compounds **40b** and **40h** exhibited strong binding affinity toward the InhA enzyme (PDB ID: 00003fne), with docking scores of −10.3 kcal/mol and −10.61 kcal/mol, respectively. These values were superior to that of the triclosan derivative 2-(2,4-dichlorophenoxy)-5-(pyridin-2-ylmethyl)phenol, which showed a docking score of −9.89 kcal/mol [55].

Additionally, the 1-methylindazole-3-carboxamide thiazolidin-4-one derivative bearing a 3-nitrophenyl group (compound **32a**) (Figure 3) exhibited favorable binding energy (ΔG = −9.99 kcal/mol) with the InhA enzyme (PDB ID: 00005jfo), along with a dissociation energy (*K*i) of 47.89 nM [62]. However, despite these promising computational results, no in vitro InhA inhibitory activity was confirmed for compound **32a**.

The two most promising triazolo [3,2-*a*][1,3,5]triazine derivatives (**62** and **64c**) exhibited good inhibitory activity against InhA, with IC_50_ values of 3.9 and 2.47 µM, respectively, compared to triclosan (IC_50_ = 1.22 µM) as a reference drug. The preferential activity of compound **64c** over compound **62** was confirmed by docking studies. Their binding energies at the active site of InhA (PDB ID: 00004dre) were −8.64 kcal/mol for compound **62**, and −12.36 kcal/mol (*E*-isomer) and −14.06 kcal/mol (*Z*-isomer) for compound **64c**. For comparison, the binding energy of the reference compound triclosan was −11.33 kcal/mol [77].

2-Acylhydrazono-5-arylidenethiazolidin-4-ones **83** and **84** (Table S1 in the Supplementary Materials) demonstrated 51% and 39% inhibition of *Mtb* InhA at a concentration of 50 µM, but were inactive against *Mtb* H_37_Rv (MIC > 25 µg/mL). However, the most active derivative, compound **61** (MIC = 6.25 µg/mL), exhibited only 22% InhA inhibition at 50 µM [57].

### 5.2. Mycobacterial Membrane Protein Large 3 (MmpL3)

Mycolic acids are long-chain fatty acids that constitute the outer layer of the mycobacterial cell wall. They protect the bacteria, contribute to drug resistance, and are essential for survival [89]. Mycobacterial membrane protein large 3 (MmpL3) is a critical transporter of mycolic acids and other lipids, indispensable for bacterial growth and viability. Due to its essential role, MmpL3 represents a promising target for novel antitubercular agents [90].

3,5-Disubstituted TZD derivatives **79g**, **79i**, and **79j** showed good binding affinity to MmpL3 (PDB ID: 00006ajj), with docking scores of −19.28 kcal/mol, −14.07 kcal/mol, and −15.63 kcal/mol, respectively (Table S1). The docking score for compound **79g**, which contains a 4-trifluoromethyl group, was slightly higher than that of the reference difluoroindole derivative ICA38 (docking score: −21.69 kcal/mol) [82]. The other 2-iminothiazolidin-4-one derivative containing a pyrrole moiety (**48a**) also demonstrated binding energy to MmpL3 (PDB ID: 00006aji) higher than that of the reference pyrrole derivative BM212 and the pyrazole derivative rimonabant, with docking scores of −9.936 kcal/mol vs. −11.11 kcal/mol and −11.59 kcal/mol, respectively [70].

### 5.3. DNA Gyrase

The series of ofloxacin-containing thiazolidin-4-one derivatives (**34a**–**34d**, **35**, and **36**) demonstrated stronger binding affinity to DNA gyrase (PDB ID: 00005bs8) in molecular docking studies than the reference drug ofloxacin (binding energy: −9.0 kcal/mol). The binding energies of compounds **34a**–**34d**, **35**, and **36** ranged from −10.7 to −10.1 kcal/mol [63]. Detailed data are presented in Table S1.

Sajid et al. conducted a theoretical analysis of the binding affinities of 22 (2-aryl-4-oxo-4,5-dihydrothiazol-5-yl)acethydrazide derivatives against DNA gyrase subunit B (PDB ID: 00003ig0), a potential target for *Mtb*. In this study, compound **85** was identified as a lead molecule, demonstrating strong binding affinity to the target protein with a docking score of −7.0 kcal/mol (Table S1) [91].

A new series of 3-(7-chloroquinolin-4-yl)aminothiazolidin-4-ones was reported as *Mtb* DNA gyrase inhibitors [64]. The most effective compounds (**37e**, **37h**, **37n**, and **38**) were evaluated for their inhibition of DNA GyrB using a DNA gyrase supercoiling assay. The results revealed that compounds **37e**, **37h**, **37n**, and **38** exhibited 16%, 36%, 68%, and 82% inhibition of DNA gyrase at a concentration of 1 µM, respectively. Molecular docking studies confirmed the potential of these four compounds as DNA gyrase inhibitors by revealing their binding affinities and interactions with the active site of GyrB ATPase (PDB ID: 00004b6c), with docking scores ranging from −4.50 to −3.14 kcal/mol (Table S1). The docking score of the most potent compound, **38** with a thiophen-2-yl substituent, correlated well with the results of the DNA supercoiling assay.

### 5.4. Other Promising Targets

Among the spirothiazolidin-4-one derivatives, compounds **44a** and **44b** exhibited an affinity for α-sterol demethylase from *Mycobacterium tuberculosis* (PDB ID: 00001ea1), with E-glide scores of −31.567 and −35.168 kcal/mol for two conformers of **44a**, and −31.696 kcal/mol for **44b** [68]. Reverse docking studies conducted during MD simulations identified stable docked poses of another spirothiazolidin-4-one compound (**43**) for the NAD-bound form (PDB ID: 00004bqp) and the apoform of InhA (PDB ID: 00004bii), as well as for Pks13 (PDB ID: 00005v3x), DprE1 (PDB ID: 00004p8c), FadD32 (PDB ID: 00005hm3), and HadAB (PDB ID: 00004rlt). Based on MM-GBSA binding energy calculations, compound 43 was found to bind with moderate affinity to HadAB (−100 to −60 kcal/mol) and the apoform of InhA (−80 kcal/mol), and with low affinity to Pks13, DprE1, FadD32, and the NAD-bound form of InhA, with binding energies ranging from −80/−70 to −60/−40 kcal/mol [67]. A detailed overview of the binding energy results is presented in Table S1.

Some 2-Iminothiazolidin-4-one derivatives demonstrated binding affinity to the MurB protein (PDB ID: 00001hsk) (compound **49c**) [71], pantothenate synthetase (PDB ID: 00003ivx) (compounds **56b**, **57a**, and **57d**) [75], and the DevR/DosR dormancy regulator (PDB ID: 00001zlk) (compounds **86**–**88**) [92].

The TZD hydroxamate derivative **77d** exhibited 41% inhibition of zinc metalloprotease 1 (Zmp1) at a concentration of 40 µM [81]. Other TZD derivatives, **69a** and **70**, demonstrated a binding affinity for β-ketoacyl-ACP synthase (KasA), with E-scores of −7.65 kcal/mol and −7.15 kcal/mol, respectively [78].

Rhodanine derivatives **80a**–**80d** (Figure 18) exhibited significant inhibitory activity against *Mycobacterium tuberculosis* protein tyrosine phosphatase B (MptpB), with IC_50_ values ranging from 0.35 to 0.64 µM. They also demonstrated acceptable selectivity, showing low inhibition against protein tyrosine phosphatase 1B (PTP1B), with IC_50_ values between 1.75 and 11.31 µM (Table S1). Additionally, compound **80c** showed notable inhibitory activity against *Mycobacterium tuberculosis* protein tyrosine phosphatase A (MptpA), with an IC_50_ of 4.06 ± 0.51 µM [58].

The rhodanine-linked enamine-carbohydrazide compound described by Maddipatla et al. demonstrated inhibitory activity against carbonic anhydrase (CA). This study evaluated CA inhibition against a panel of human CA isoforms I and II, as well as *Mycobacterium tuberculosis* carbonic anhydrase isoforms (mtCA 1–3). The compound showed only moderate inhibition of human CA isoforms. In contrast, it exhibited stronger inhibitory potential against the *Mycobacterium tuberculosis* isoforms mtCA1, mtCA2, and mtCA3. Among the series, compound **89**, bearing a 4-nitro group, was the most potent inhibitor of mtCA2, with a *K*i value of 9.5 µM (Table S1). However, it displayed only moderate activity against *Mtb* H37Rv, with an MIC of 32 µg/mL. Interestingly, compound **82**, which was the most effective against *Mtb* H37Rv (MIC = 1 µg/mL), exhibited a similar binding mode to that of compound **89** with mtCA2. Both compounds **82** and **89** demonstrated metal coordination with the zinc ion, a characteristic feature of classical carbonic anhydrase inhibitors. These compounds demonstrated selectivity toward mtCA2, as indicated by the screening results [83].

In the search for new therapeutic agents against *Mycobacterium tuberculosis*, several thiazolidin-4-one derivatives have shown promising activity against key *Mtb* targets involved in essential metabolic and structural pathways. These targets include InhA, MmpL3, DNA gyrase, and other enzymes related to cell wall biosynthesis, lipid transport, and bacterial survival mechanisms. Multiple compounds demonstrated strong binding affinity in molecular docking studies and, in some cases, notable in vitro inhibitory activity. Particularly effective derivatives included those targeting InhA (e.g., **40b**, **40h**, and **64c**), MmpL3 (e.g., **79g**), and DNA gyrase (e.g., **38**). Moreover, select compounds showed specificity for *Mtb* enzymes over human homologs, such as compound **89** targeting mtCA2. Overall, these findings support the potential of thiazolidinone-based scaffolds as versatile platforms for the development of novel antitubercular agents.

## 6. Conclusions and Future Perspectives

In conclusion, thiazolidin-4-one derivatives have emerged as a versatile class of compounds with significant potential in antitubercular drug discovery, demonstrating potent activity against drug-sensitive, multidrug-resistant, and extensively drug-resistant *Mycobacterium tuberculosis* strains. Their structural flexibility allows for diverse chemical modifications that enhance efficacy, selectivity, and target specificity, as shown by structure–activity relationship studies and in vitro assays. Many compounds have shown favorable cytotoxicity profiles and multitarget activity, inhibiting key enzymes such as InhA, MmpL3, and DNA gyrase. Future perspectives should focus on the optimization of lead compounds through SAR and QSAR modeling, comprehensive in vivo pharmacokinetic and toxicity profiling, and the evaluation of synergistic effects in combination with existing antitubercular therapies. Further mechanistic studies employing crystallography and molecular dynamics are needed to confirm binding modes, while the design of dual- or multi-target hybrid molecules could provide new solutions to overcome resistance and improve therapeutic efficacy.

## Figures and Tables

**Table 1:** Antitubercular activity of compounds **40a**–**40m**.

Compound	*Mycobacterium tuberculosis* Strains, MIC (µg/mL)		
	Drug-Sensitive H_37_Ra	MDR	XDR
**40a**	62.5	>125	na
**40b**	0.48	3.9	31.25
**40c**	31.25	na	na
**40d**	15.63	na	na
**40e**	1.95	15.63	>125
**40f**	15.63	125	na
**40g**	7.81	62.5	na
**40h**	0.12	1.95	7.81
**40i**	0.98	15.63	125
**40j**	3.9	31.25	na
**40k**	7.81	125	na
**40l**	3.9	62.5	na
**40m**	0.48	7.81	15.63
isoniazid	0.12	na	na

MIC—minimum inhibitory concentration; MDR—multidrug-resistant strain; XDR—extensively drug-resistant strain; na—inactive compound.

## Data Availability

Data are contained within the article and Supplementary Materials.

## Supplementary Materials

The following supporting information can be downloaded at https://www.mdpi.com/article/10.3390/molecules30102201/s1, Table S1: Potential molecular targets of thiazolidin-4-one derivatives with corresponding in vitro activity and computational binding data.

## Abbreviations

The following abbreviations are used in this manuscript:

AMP	adenosine monophosphate
ATP	adenosine triphosphate
CA	carbonic anhydrase
Dev/DosR	DevR/DosR dormancy regulator
DMAD	dimethyl acetylenedicarboxylate
DNA	deoxyribonucleic acid
DprE1	decaprenylphosphoryl-β-D-ribose 2′-oxidase
FadD32	fatty acid degradation protein D32
FICI	fractional inhibitory concentration index
HadAB	(3R)-hydroxyacyl-ACP dehydratase heterodimer
HEK-293	human embryonic kidney cell line
HIV	human immunodeficiency virus
IC_50_	half maximal inhibitory concentration
InhA	enoyl-acyl carrier protein reductase
KasA	β-ketoacyl-acyl carrier protein synthase
*K*i	inhibition constant
MDR	multidrug-resistant
MIC	minimum inhibitory concentration
MmpL3	mycobacterial membrane protein large 3
MptpA	*Mycobacterium tuberculosis* protein tyrosine phosphatase A
MptpB	*Mycobacterium tuberculosis* protein tyrosine phosphatase B
*Mtb*	*Mycobacterium tuberculosis*
MurB	UDP-*N*-acetylenolpyruvoylglucosamine reductase
Pks13	polyketide synthase
PS	pantothenate synthetase
PTP1B	protein tyrosine phosphatase 1B
QSAR	quantitative structure–activity relationship
RAW 264.7	adherent cell line isolated from a mouse tumor that was induced by Abelson murine leukemia virus
SAR	structure–activity relationship
TB	tuberculosis
THF	tetrahydrofuran
TZD	thiazolidine-2,4-dione
XDR	extensively drug-resistant
Zmp1	zinc metalloprotease 1
