# Supplementary material for: Review of Recent Advances in Thiazolidin-4-One Derivatives as Promising Antitubercular Agents (2021–Present)
Source: Molecules. 2025 May 17;30(10):2201. doi: 10.3390/molecules30102201 (PMC12114044; doi:10.3390/molecules30102201)
Supplement: Supplementary file 1 [file molecules-30-02201-s001.zip › molecules-3632423-supplementary.pdf]

Supplementary materials for

Review of recent advances in thiazolidin-4-one derivatives as promising antitubercular agents (2021–present)

Wiktoria Drzał [1] and Nazar Trotsko [2,*]

[1] Department of Organic Chemistry, Students Research Group, Medical University of Lublin, 4A Chodzki Street, 20-093 Lublin, Poland; 63752@student.umlub.pl (W.D.)
[2] Department of Organic Chemistry, Medical University of Lublin, 4A Chodzki Street, 20-093 Lublin, Poland; nazar.trotsko@umlub.pl (N.T.)
* Correspondence: nazar.trotsko@umlub.pl (N.T.)

Table S1. Potential molecular targets of thiazolidin-4-one derivatives with corresponding *in vitro* activity and computational binding data.

Compound	Structure	Target	*In vitro* activity results	Computational binding results	References
**52a**	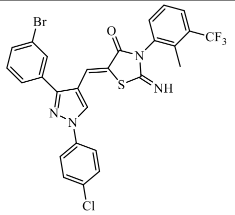	enoyl-acyl carrier protein reductase (InhA)	-	Docking score: −6.41 kcal/mol (InhA, PDB ID: 00001p44)	[71]
**40b**	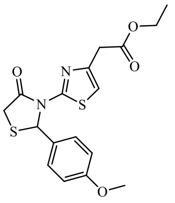	enoyl-acyl carrier protein reductase (InhA)	IC_{50} = 1.3±0.61 μM	Docking score: −10.3 kcal/mol (InhA, PDB ID: 00003fne)	[62]
**40h**	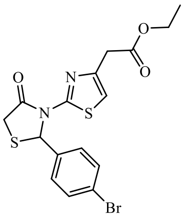	enoyl-acyl carrier protein reductase (InhA)	IC_{50} = 1.06±0.97 μM	Docking score: −10.61 kcal/mol (InhA, PDB ID: 00003fne)	[62]
**32a**	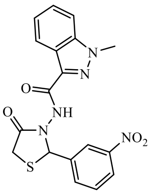	enoyl-acyl carrier protein reductase (InhA)	-	ΔG = −9.99 kcal/mol Ki = 47.89 nM (InhA, PDB ID: 00005jfo)	[57]
**62**	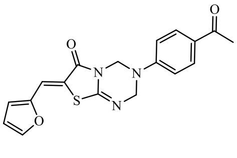	enoyl-acyl carrier protein reductase (InhA)	IC_{50} = 3.9±0.09 μM	Docking score: −8.64 kcal/mol (InhA, PDB ID: 00004dre)	[76]
**64c**	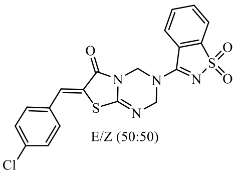	enoyl-acyl carrier protein reductase (InhA)	IC_{50} = 2.47±0.11 μM	Docking score: −12.36 kcal/mol (isomer *E*) (InhA, PDB ID: 00004dre) Docking score: −14.06 kcal/mol (isomer *Z*) (InhA, PDB ID: 00004dre)	[76]
**83**	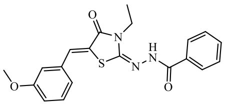	enoyl-acyl carrier protein reductase (InhA)	51% inhibition at 50 μM	-	[75]
**84**	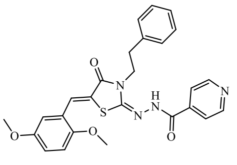	enoyl-acyl carrier protein reductase (InhA)	39% inhibition at 50 μM	-	[75]

**79g**	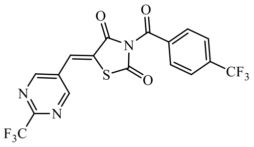	mycobacterial membrane protein large 3 (MmpL3)	-	Docking score: −19.28 kcal/mol (MmpL3, PDB ID: 00006ajj)	[81]
**79i**	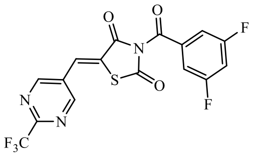	mycobacterial membrane protein large 3 (MmpL3)	-	Docking score: −14.07 kcal/mol (MmpL3, PDB ID: 00006ajj)	[81]
**79j**	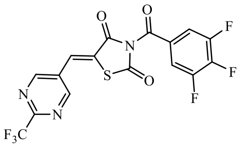	mycobacterial membrane protein large 3 (MmpL3)	-	Docking score: −15.63 kcal/mol (MmpL3, PDB ID: 00006ajj)	[81]
**48a**	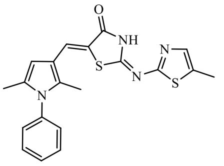	mycobacterial membrane protein large 3 (MmpL3)	-	Docking score: −9.936 kcal/mol (MmpL3, PDB ID: 00006aji)	[68]
**34a**	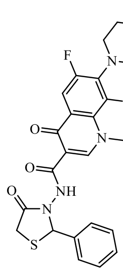	DNA Gyrase	-	Binding energy: −10.5 kcal/mol (DNA Gyrase, PDB ID: 00005bs8)	[59]
**34b**	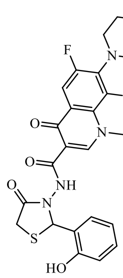	DNA Gyrase	-	Binding energy: −10.5 kcal/mol (DNA Gyrase, PDB ID: 00005bs8)	[59]
**34c**	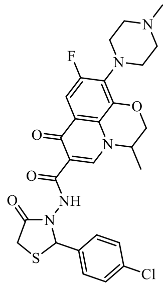	DNA Gyrase	-	Binding energy: −10.7 kcal/mol (DNA Gyrase, PDB ID: 00005bs8)	[59]
**34d**	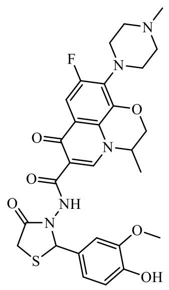	DNA Gyrase	-	Binding energy: −10.6 kcal/mol (DNA Gyrase, PDB ID: 00005bs8)	[59]

**35**	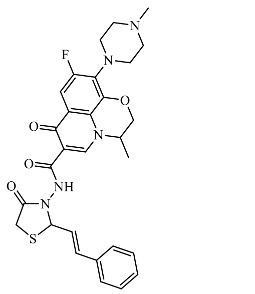	DNA Gyrase	-	Binding energy: −10.7 kcal/mol (DNA Gyrase, PDB ID: 00005bs8)	[59]
**36**	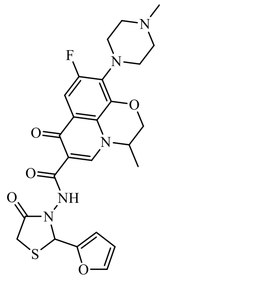	DNA Gyrase	-	Binding energy: −10.1 kcal/mol (DNA Gyrase, PDB ID: 00005bs8)	[59]
**85**	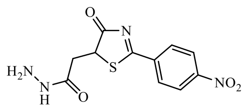	DNA Gyrase	-	Binding affinity: −7.0 kcal/mol (GyrB, PDB ID: 00003ig0)	[91]
**37e**	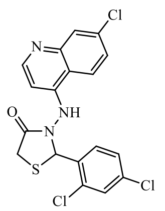	DNA Gyrase	DNA supercoiling assay: 16% inhibition at 1 μM	Docking score: −3.945 kcal/mol (GyrB ATPase, PDB ID: 00004b6c)	[60]
**37h**	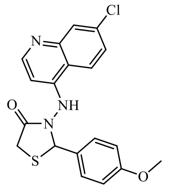	DNA Gyrase	DNA supercoiling assay: 36% inhibition at 1 μM	Docking score: −4.437 kcal/mol (GyrB ATPase, PDB ID: 00004b6c)	[60]
**37n**	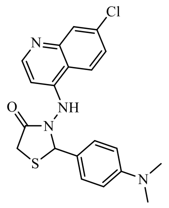	DNA Gyrase	DNA supercoiling assay: 68% inhibition at 1 μM	Docking score: −3.14 kcal/mol (GyrB ATPase, PDB ID: 00004b6c)	[60]
**38**	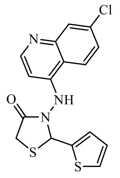	DNA Gyrase	DNA supercoiling assay: 82% inhibition at 1 μM	Docking score: −4.496 kcal/mol (GyrB ATPase, PDB ID: 00004b6c)	[60]
**44a**	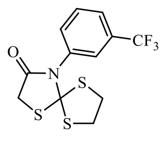	*α*-sterol demethylase (CYP51)	-	E-glide: −31.567 kcal/mol and −35.168 kcal/mol (CYP51, PDB ID: 00001ea1) two conformers	[65]

**44b**	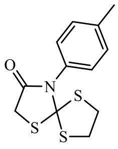	α-sterol demethylase (CYP51)	-	E-glide: −31.696 kcal/mol (CYP51, PDB ID: 00001ea1)	[65]
**43**	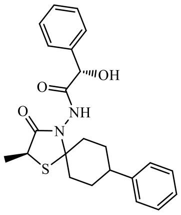	NAD-bound form of InhA	-	MM-GBSA binding energy: −60/−40 kcal/mol (InhA, PDB ID: 00004bqp)	[64]
		Apoform of InhA		MM-GBSA binding energy: −80 kcal/mol (InhA, PDB ID: 00004bii)	[64]
		Pks13		MM-GBSA binding energy: −65/−45 kcal/mol (Pks13, PDB ID: 00005v3x)	[64]
		DprE1		MM-GBSA binding energy: −70/−55 kcal/mol (DprE1, PDB ID: 00004p8c)	[64]
		FadD32		MM-GBSA binding energy: −80/−70 kcal/mol (FadD32, PDB ID: 00005hm3)	[64]
		HadAB		MM-GBSA binding energy: −100/−60 kcal/mol (HadAB, PDB ID: 00004rlt)	[64]
**49c**	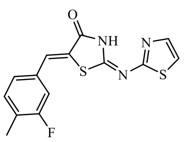	MurB protein	-	Binding affinity: −9.4 kcal/mol (MurB, PDB ID: 00001hsk)	[69]
**56b**	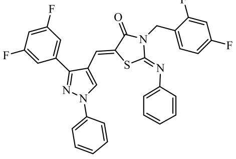	pantothenate synthetase (PS)	-	Binding affinity: −10.0 kcal/mol (PS, PDB ID: 00003ivx)	[73]

**57a**	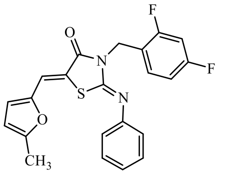	pantothenate synthetase (PS)	-	Binding affinity: −10.1 kcal/mol (PS, PDB ID: 00003ivx)	[73]
**57d**	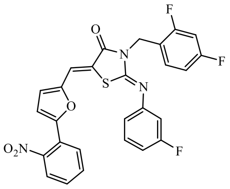	pantothenate synthetase (PS)	-	Binding affinity: −10.1 kcal/mol (PS, PDB ID: 00003ivx)	[73]
**86**	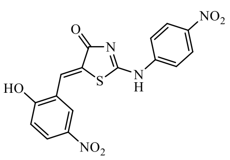	DevR/DosR dormancy regulator	-	Binding energy: −5.45 kcal/mol (chain A) −5.86 kcal/mol (chain B) (DevR, PDB ID: 00001zlk)	[92]
**87**	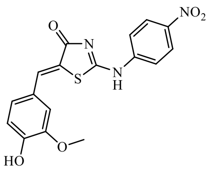	DevR/DosR dormancy regulator	-	Binding energy: −5.45 kcal/mol (chain A) −7.16 kcal/mol (chain B) (DevR, PDB ID: 00001zlk)	[92]
**88**	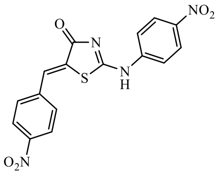	DevR/DosR dormancy regulator	-	Binding energy: −5.16 kcal/mol (chain A) −5.73 kcal/mol (chain B) (DevR, PDB ID: 00001zlk)	[92]
**77d**	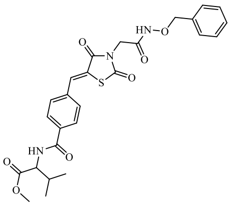	zinc metallo-protease 1 (Zmp1)	41±9% inhibition of Zmp1 at 40 μM	-	[80]
**69a**	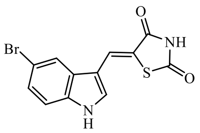	β-ketoacyl-ACP synthase (KasA)	-	Docking score: −7.65 kcal/mol (KasA, PDB ID: 00002wge)	[77]
**70**	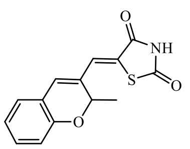	β-ketoacyl-ACP synthase (KasA)	-	Docking score: −7.15 kcal/mol (KasA, PDB ID: 00002wge)	[77]

**80a**	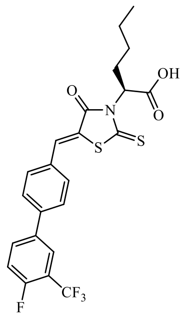	MptpB<br>PTP1B<br>MptpA	IC_{50} = 0.48±0.08 μM<br>IC_{50} =11.31 μM<br>20.16% inhibition at 10 μM	-	[82]
**80b**	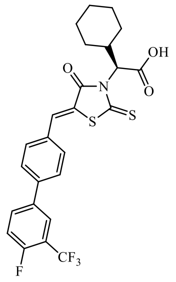	MptpB<br>PTP1B<br>MptpA	IC_{50} = 0.49±0.06 μM<br>IC_{50} = 5.37 μM<br>27.13% inhibition at 10 μM	-	[82]
**80c**	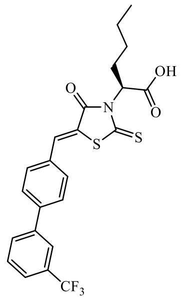	MptpB<br>PTP1B<br>MptpA	IC_{50} = 0.64±0.04 μM<br>IC_{50} = 4.34 μM<br>IC_{50} = 4.06±0.51 μM	-	[82]
**80d**	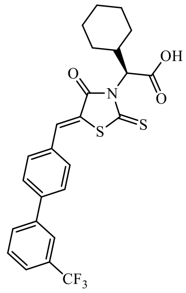	MptpB<br>PTP1B<br>MptpA	IC_{50} = 0.35±0.03 μM<br>IC_{50} = 1.75 μM<br>16.65% inhibition at 10 μM	-	[82]
**89**	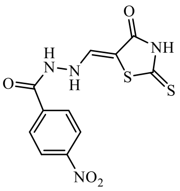	*Mtb* carbonic anhydrase isoform 2 (mtCA 2)	*K*i = 9.5 μM	-	[83]
**82**	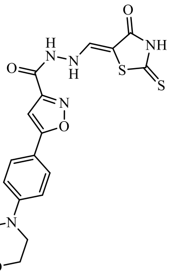	*Mtb* carbonic anhydrase isoform 2 (mtCA 2)	*K*i = 63.9 μM	-	[83]
